# MicroCT Can Characterize Clots Retrieved With Mechanical Thrombectomy From Acute Ischemic Stroke Patients–A Preliminary Report

**DOI:** 10.3389/fneur.2022.824091

**Published:** 2022-03-07

**Authors:** Daniela Dumitriu LaGrange, Vincent Braunersreuther, Isabel Wanke, Jatta Berberat, Siri Luthman, Seán Fitzgerald, Karen M. Doyle, Olivier Brina, Philippe Reymond, Alexandra Platon, Michel Muster, Paolo Machi, Pierre-Alexandre Poletti, Maria Isabel Vargas, Karl-Olof Lövblad

**Affiliations:** ^1^Division of Diagnostic and Interventional Neuroradiology, HUG Geneva University Hospitals, Geneva, Switzerland; ^2^Division of Clinical Pathology, Diagnostic Department, HUG Geneva University Hospitals, Geneva, Switzerland; ^3^Division of Neuroradiology, Klinik Hirslanden, Zurich, Switzerland; ^4^Swiss Neuroradiology Institute, Zurich, Switzerland; ^5^Division of Neuroradiology, University of Essen, Essen, Germany; ^6^Department of Psychiatry, University of Geneva, Geneva, Switzerland; ^7^Division of Neuroradiology, Kantonsspital Aarau, Aarau, Switzerland; ^8^Department of Physiology, National University of Ireland, Galway, Ireland; ^9^CÚRAM, Science Foundation Ireland (SFI), Centre for Research in Medical Devices, National University of Ireland Galway, Galway, Ireland; ^10^Division of Radiology, HUG Geneva University Hospitals, Geneva, Switzerland

**Keywords:** clot, acute ischemic stroke, CT density, red blood cells, fibrin

## Abstract

**Background:**

Characterization of the clot occluding the arteries in acute ischemic stroke received ample attention, in terms of elucidating the relationship between the clot composition, its etiology and its amenability for pharmacological treatment and mechanical thrombectomy approaches. Traditional analytical techniques such as conventional 2D histopathology or electron microscopy sample only small parts of the clot. Visualization and analysis in 3D are necessary to depict and comprehend the overall organization of the clot. The aim of this study is to investigate the potential of microCT for characterizing the clot composition, structure, and organization.

**Methods:**

In a pilot study, we analyzed with microCT clots retrieved from 14 patients with acute ischemic stroke. The following parameters were analyzed: overall clot density, clot segmentation with various density thresholds, clot volume.

**Results:**

Our findings show that human clots are heterogeneous in terms of CT intra-clot density distribution. After fixation in formalin, the clots display a shift toward negative values. On average, we found the mean HU values of red clots retrieved from patients to be −153 HU, with SD = 23.8 HU, for the intermediate clots retrieved from patients −193 HU, SD = 23.7 HU, and for the white clots retrieved from patients −229 HU, SD = 64.8 HU.

**Conclusion:**

Our study shows that volumetric and density analysis of the clot opens new perspectives for clot characterization and for a better understanding of thrombus structure and composition.

## Introduction

The composition of occlusive clots in acute ischemic stroke has received vast attention through two dimensional (2D) histopathology investigations (1–3). Knowledge about human clots composition allowed valuable developments regarding the preparation of clot analogs (4) which best reproduce in terms of cellular and fibrin content the composition of clots retrieved from patients. Clot analogs with predefined compositions were successfully used to define the most effective clinical imaging protocols, computed tomography (CT) (5) or magnetic resonance imaging (MRI) (6), or to study *in vitro* thrombectomy techniques (4). While clot analogs are a valuable tool for studying clinical imaging and optimizing interventional techniques, the clots retrieved from patients remain the most valuable substrate for research on clot behavior (7). Traditional techniques, such as conventional 2D histopathology and electron microscopy, provide a wealth of information on clot composition, etiology and markers associated with resistance to treatment (8, 9). These techniques, which often sample small representative areas of the clot, provide qualitative information. However, techniques suitable for retrieving quantitative information about the structure and organization of human clots are underdeveloped. When available, such techniques could help establishing meaningful correlations between the clot characteristics and clinical aspects in stroke. Volumetric analysis of the clot is necessary in order to gather quantitative information. Emerging approaches in the field of three dimensional (3D) visualization of clots are fluorescent labeling of clots after optical clearing (10), or integration of signals provided by optical coherence tomography (11). X-rays based imaging techniques, such as microCT, although they have been extensively used for various tissues characterization (12, 13), are still underappreciated in terms of clot characterization.

In our study we perform a preliminary investigation into the potential that microCT holds for characterizing clots retrieved from patients with acute ischemic stroke. We illustrate that clots retrieved from patients vary in terms of CT density, which can be correlated with the clot composition, and display a heterogenous distribution of CT density.

## Methods

We included in the study 14 patients (7 males, 7 females, age 41–95 years) who were referred to our institution suffering acute stroke, underwent dual-source CT in the emergency department, and did not qualify for thrombolytic treatment prior thrombectomy. Thrombectomy was performed according to the standard clinical practice.

In addition, three types of clot analogs, prepared according to the protocols described elsewhere (4), were made available for imaging with microCT in our study.

### MicroCT Imaging

*Ex vivo* experiments were carried out on a low dose X-Ray micro computed tomography scanner (Quantum GX, Perkin Elmer). The scanner uses a cone beam X-ray source and a flat panel X-ray detector to acquire high quality slice images, which are rendered for 3D visualization. The detector was calibrated as such that Hounsfield Units (HU) values were adjusted for air (−1,000) and water (0). Clots were imaged upon placing them in a sealed falcon tube, to maintain moisture and prevent tissue degradation prior to imaging, either as retrieved from patient (fresh) before and after rinsing with saline solution NaCl 0.9%, or after fixation in formalin. Each formalin-fixed clot was rinsed with saline solution, drained on sterile pads and subsequently placed in the sealed falcon tube. The clots were imaged with the low noise imaging protocol (14 min), with 90 kV X-ray energy, 88 μA, and 50 μm voxel size. Segmentation and quantification of clots attenuation *ex vivo* was performed in 3D Slicer^1^ (14).

### Histopathology

Histology staining was performed after embedding the formalin-fixed clots in paraffin, on 3 micrometers thick slices, according to standard protocols (1) with Martius Scarlet Blue (MSB) and Hematoxylin and Eosin (HE).

### Statistical Methods

The statistics were calculated using SPSS version 25 (SPSS Inc., Chicago, IL, USA). The normality of data distribution was assessed with Shapiro-Wilk test. The relationships between variables (retrieved clot volume and volume of dense part of the clot, average density of the retrieved clot, and retrieved clot volume) were examined using the Spearman rank correlation test.

## Results

### Patients Characteristics

We included in this preliminary study only patients which did not qualify for thrombolytic therapy. Baseline characteristics are presented in Table 1. The table includes information on the antithrombotic medication prior to thrombotic event, time elapsed from the thrombotic event (when > 4.5 h), conditions contraindicating the thrombolytic therapy, thrombectomy interventions and outcome.

**Table 1:** Baseline characteristics of patients with acute ischemic stroke included in this pilot study.

	**Retrieved clot volume, V (mm^3^)**		
	**V <25**	**25 < V <55**	**V > 55**
**Patients**, ***n***	4	4	6
**Age (years), mean**	70	63	77
**Gender (male)**, ***n***	2	3	2
Antithrombotic medications^*^, ***n***			
Antiplatelet	-	1	1
Anticoagulant	3	2	3
Antiplatelet and anticoagulant	1	-	1
**Time from thrombotic event onset -to-treatment** **>4.5 h**, ***n*** (mean, h)^**^	2 (14 h)	2 (7 h)	6 (14 h)
**Conditions contraindicating the thrombolytic therapy**, ***n***			
Arterial hypertension	1	2	2
**Occlusion location**, ***n***			
M1	3	3	4
M2	1	1	1
P4	-	-	1
**Type of clot**, ***n***			
White	2	-	-
Intermediate	2	1	1
Red	-	3	5
**Endovascular technique**, ***n***			
Aspiration	3	1	2
Stent retriever	-	-	-
Combination	3	4	6
**No. of passes, mean (min, max)**	3.3 (1,6)	2.8 (1,4)	2.8 (1,5)
**Final TICI score**, ***n***			
1	1	-	1
2b	-	-	2
2c	1	1	-
3	2	3	3

* *Medication received at the time of thrombotic event.*

** *When time from thrombotic event onset-to-treatment > 4.5 h*.

### Imaging Clot Analogs and Clots Retrieved From Patients With Acute Ischemic Stroke

**Figure 1:**
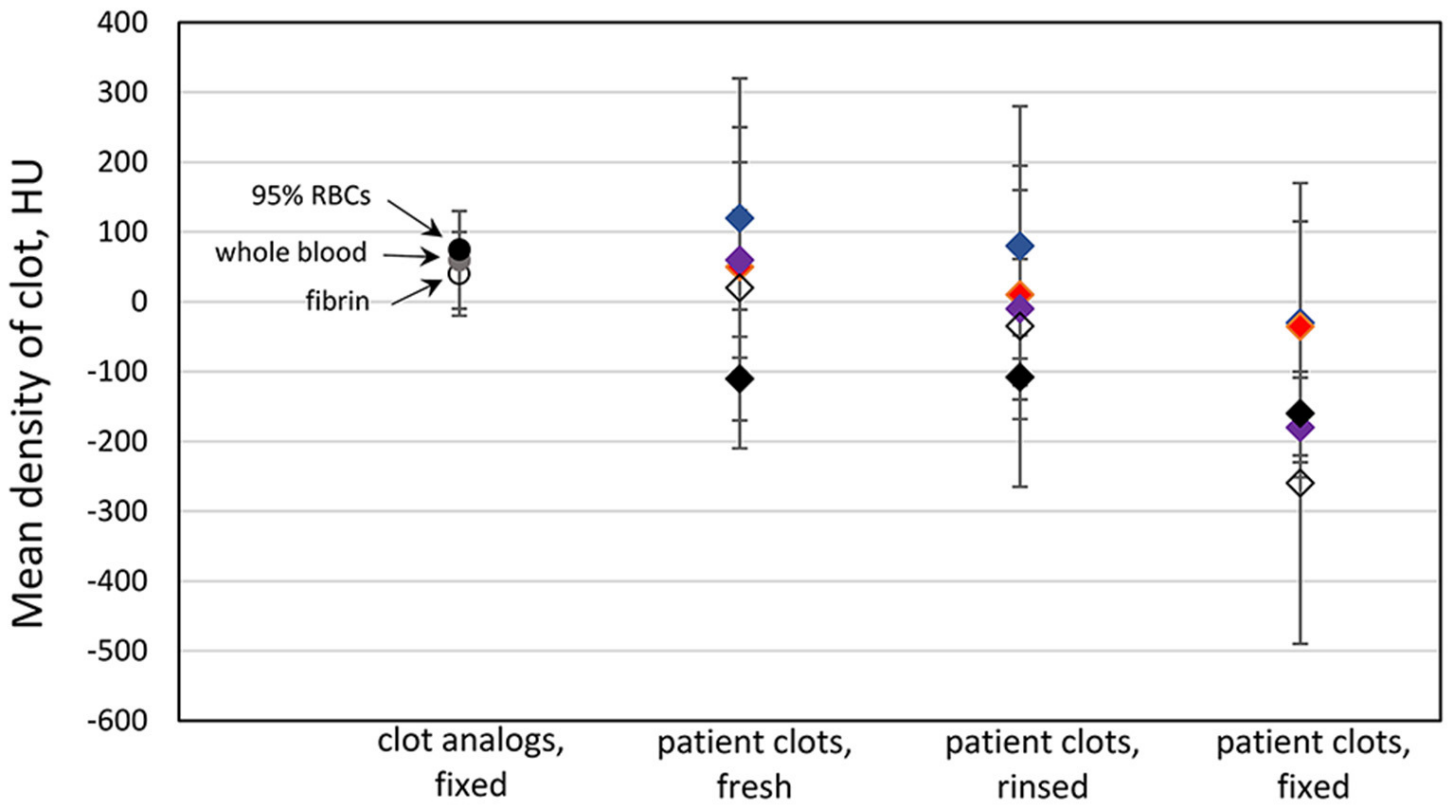
Clot density in microCT—comparison of clot analogs and human clots at different processing stages. Data points represent the mean HU values for each clot, and the bars indicate the standard deviation of the density distribution within each clot. Same color of diamond shape data points was used for each individual clot. The clots were imaged in three states: fresh, rinsed in saline, and after being fixed in formalin.

Our results are showing that clots retrieved from patients are more diverse in terms of CT density, and display a widespread distribution, compared to clot analogs with pre-established compositions (Figure 1). We observed a systematic decrease in clot density, measured in HU, from fresh (as collected) state, after rinsing with saline solution, and further after fixation in formalin. The mean HU values for human formalin-fixed clots are in negative range. After fixation in formalin, we found an average of mean HU values within red clots group equal to −153 HU, with standard deviation (SD) = 23.8 HU, an average of mean HU within intermediate clots group −193 HU, SD = 23.7 HU, and an average of mean HU within white clots group −229 HU, SD = 64.8 HU. For each clot, no differences were observed in CT density regardless the fixation time (2 h, 6 h, or 3 days), which indicates an initial stabilization in the structural features which are detected with CT, within the first 2 h of fixation in formalin.

**Figure 2:**
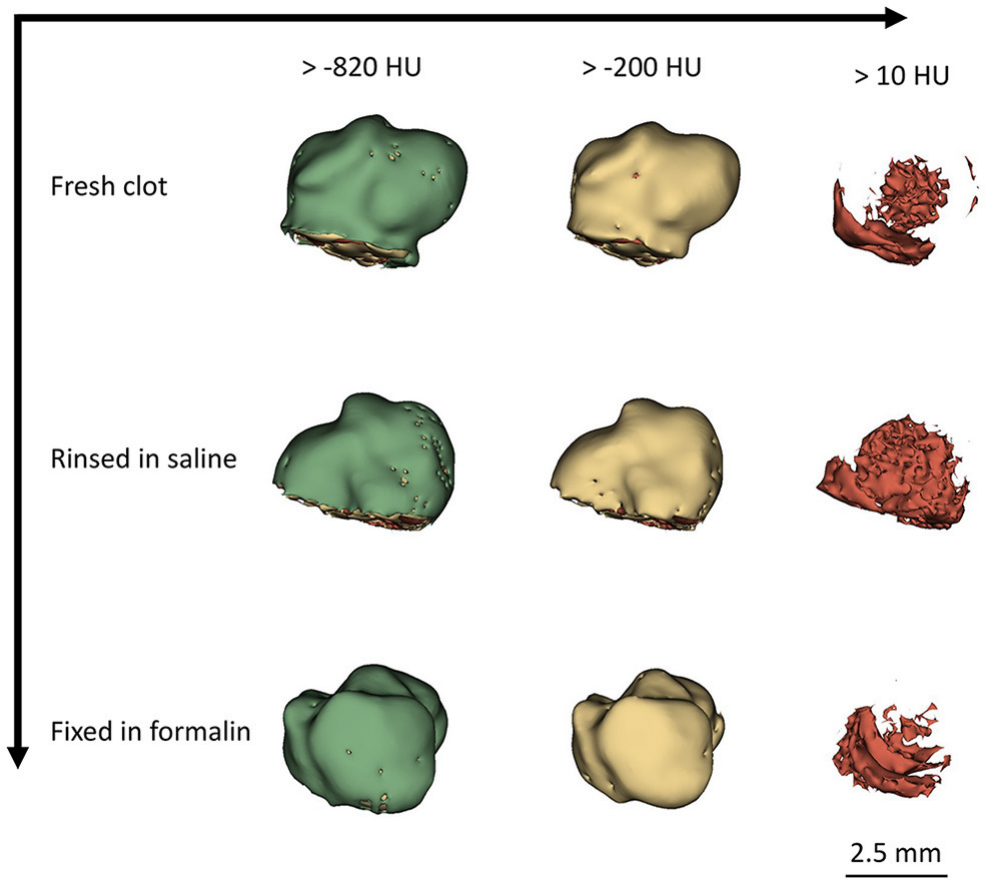
Segmentation of clot at different processing stages.

The microCT appearance of human clots is highly heterogeneous. Typically, a thin region (100–200 micrometers) of low density (−850 to −200 HU) is associated with the partial filling of the voxels at the outer surface of the sample, most likely related with a discontinuous layer of plasma proteins and water (serum) in fresh samples and, especially, in fixed samples, with the surface roughness. An example is illustrated in Figure 2. We observe the presence of dense regions (for which we defined a threshold of 10 HU, density values above those of water range), persistent throughout rinsing and fixing in formalin (Figure 2).

**Figure 3:**
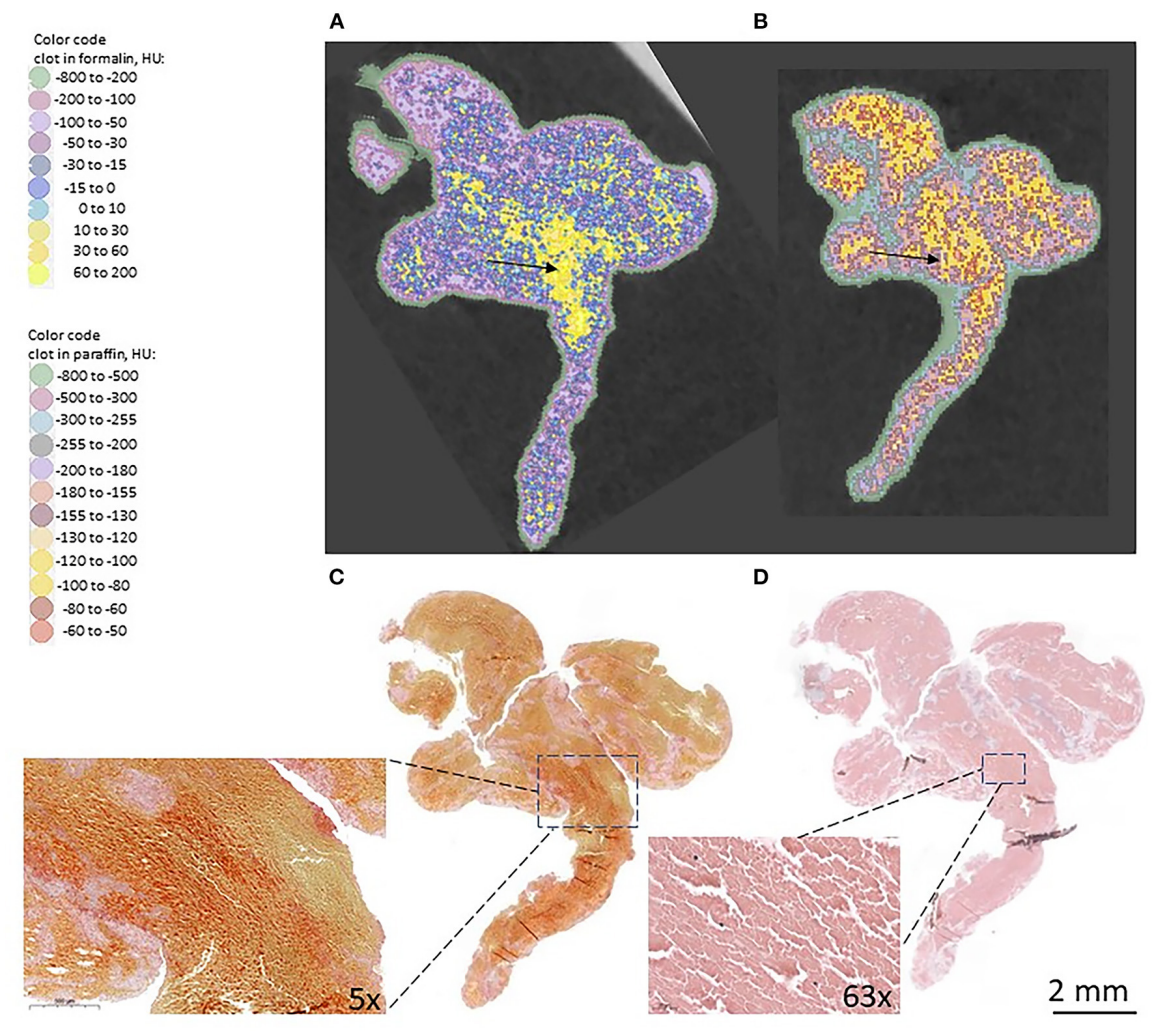
Comparison microCT and histological staining: **(A)** microCT slice of formalin-fixed clot; **(B)** microCT slice of clot embedded in paraffin; **(C)** Histological staining of the clot with Martius Scarlet Blue (MSB), in which RBCs appear yellow and fibrin appears red; **(D)** Histological staining of the clot with hematoxylin and eosin (HE). In the inset: RBCs rich region.

In a first attempt to investigate how the microCT features relate to the histology of the clot, we performed a side-by-side comparison with histological staining (Figure 3), and information included in Supplemental Material. In absence of 3D histological rendering, an unequivocal assignment of histological features to CT density ranges is not possible. Only a qualitative interpretation of the information rendered by the two techniques, using 2D depiction, can be performed. As such, our data suggest that red blood cells (RBCs) can span a wide range in density (-200 to 200 HU), which is partially overlapped by the density of water (−30 to 10 HU, blue color code in Figure 3A). We observed that in the RBCs rich sample the highest density regions (> 10 HU, above water density) for the clot in wet state (Figure 3A) do translate in the paraffin embedded sample also as relatively augmented CT density regions (Figure 3B) and correspond to compact regions of RBCs in histologically stained slices (Figures 3C,D; Supplemental Material). However, in clots with lower concentrations of RBCs, such as intermediate clots, as presented in Supplementary Figures 1, 2, the regions that appear most dense in wet state fade in density after embedding the clot in paraffin. Only a partial overlap can be observed between the densest regions in wet state with the RBCs agglomerates observed in histological staining.

Platelets and fibrin regions, as identified by histological staining, consistently appear in microCT of fixed samples in low density regions (water density or below) and in paraffin embedded samples overlap, for the most part, with the density of paraffin (−500 to −180 HU, mean 345 HU).

Heterogeneity in various tissues can be analyzed through textural features computed from CT scans (15). Similar algorithms can be applied for analyzing clots retrieved from patients. As an example, maps computed for gray level co-occurrence matrices (GLCM) and gray-level run length matrices (GLRLM) for a retrieved clot are presented in Supplementary Figure 3.

**Figure 4:**
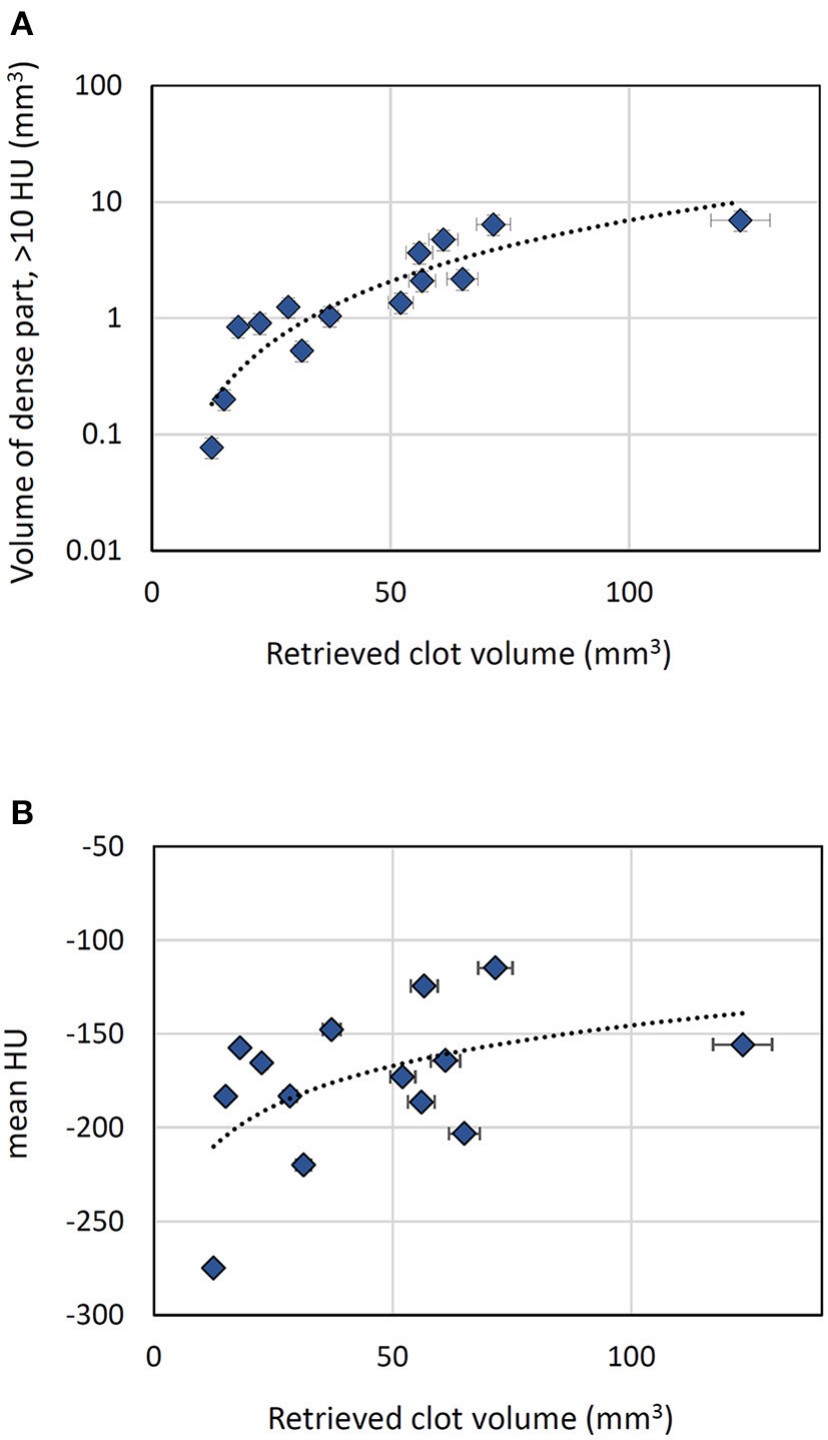
Volumetric analysis of the retrieved clots (data points refer to formalin-fixed clots). **(A)** Correlation between the volume of dense part of the clot and the overall clot volume. **(B)** Variation of clot average density (mean HU) with the clot volume.

We studied the volumetric CT density distribution of the clots included in our study. In brief, the average volumetric content in regions with density > 10 HU (v_d_ %) is changing as following: 5.34 vol%, with SD = 2.31 vol% for red clots, 3.35 vol%, with SD = 1.19 vol% for intermediate clots, and 0.98 vol% with SD = 0.50 vol% for white clots. We also examined the relation between the volume of dense regions (v_d_, with >10 HU, above the density of water) and the overall volume of the clot. We found a large positive relationship between the two sets of values, with a Spearman correlation factor r(12) = 0.943 (two-tailed *p* < 0.001) (Figure 4A). This finding is not surprising, since we observed larger retrieved clot volumes for red clots, compared to the white clots. Contrasting to this finding, we identified only a non-significant medium positive association between the mean HU values of each overall clot and the clot volume [r(12) = 0.4593, two-tailed *p* value = 0.1043] (Figure 4B), which we consider an indicator for the non-uniform contribution of the porosity to the overall density values of the clot.

## Discussion

Occlusive clots causing ischemic stroke, including both thrombi and emboli, form under complex blood flow conditions and have various etiologies. Therefore, the clots retrieved from patients differ from the clot analogs and vary with respect to each other in terms of composition, overall porosity, size, distribution of cellular aggregates, and fibrin organization.

Our study shows that microCT can capture information regarding the heterogeneous intra-clot distribution of density, which we illustrate with segmentations performed for various density ranges. We exemplify that through volumetric analysis of clot CT density we can define quantitative variables which can be correlated with clot organization. A limitation of this study is the small sample size, which does not allow performing correlative links between the textural features and clot composition.

A drawback of the study is a limited access to fresh clots. More research is necessary to elucidate how interstitial blood and residual contrast agent are contributing to the CT density of fresh clots, as retrieved. The CT imaging of fixed clots is most likely devoid of such additional contributions. CT imaging of human clots has the potential to be rendered relevant for histological features of the clot. Imaging fresh clots could become a valuable characterization technique prior to biomechanical testing.

The shift toward negative density values after fixing the clot in formalin is not surprising. We consider it a consequence of the clot dehydration due to the fixation process, which results in hydrophobic effects that trigger an increase in porosity of the overall clot, once removed from formalin solution.

The preparation conditions for histological staining involve some degree of distortion of the original histological material, and a direct compositional correlation between histology slides renderings and microCT cross sectional view is difficult. The microCT slices have a thickness (50 micrometers) much larger than the thickness of the clot slices used for histological staining (3 micrometers). Therefore, the microCT slices integrate more information related to the composition and structure of the clot, compared to the information rendered by each individual histological slide. Especially in small clots with intermediate composition, for which the size of compositionally distinct regions is small, establishing a correspondence between the histological staining and microCT density is unlikely when resolution limits are at play and partial filling effects of individual voxels with different histological structures and media (water or paraffin) occur. However, we still consider the highest density range observed for the clots included in our study (>10 HU and up to 200 HU in wet samples) as being related to the compactness of the clot in preferential regions, most likely with the presence of RBCs agglomerates. We rule out the presence of calcifications as being responsible for the above-mentioned density range (10–200 HU). Calcifications are not specific for red and intermediate clots (16, 17), and, as described in Supplementary Figure 4, the density we observe for calcifications having similar size and distribution as the dense (typically 10–200 HU) regions in red clots is much higher (1,000 HU and above).

Arterial, red clots in acute ischemic stroke are often characterized by core regions of compact RBCs, also known as polyhedrocites, while the outer regions are formed by biconcave RBCs and fibrin (8). Agglomerates of compact RBCs are a marker for clot intravital contraction (8, 18–20). While the clinical implications of clot contraction in acute ischemic stroke remain to be elucidated, it has been hypothesized that clot contraction can be an underlying mechanism that reduces vessel occlusion and allows blood flow past clot (21). MicroCT could be, for example, useful in quantifying the degree of clot contraction and in exploring how clot contraction affects the clinical manifestation of stroke.

Within the limitations of our study, we showed that an attribution of the dense regions found in microCT of fixed clots to RBCs rich compact regions is reasonable. This remains a strong hypothesis, to be verified by future studies, which will be using fiducials to compare microCT volumetric renderings with serial 3D histopathology findings. While handling fresh clots for microCT characterization can prove to be difficult, due to perishability of fresh samples and limited sample availability during normal working hours when microCT operation is possible, handling formalin-fixed samples is much more convenient. A large sample size of clots, measured in fresh and in fixed state, might provide standardized means to best exploit the information gathered from CT characterization of clots retrieved from patients.

Our study shows that volumetric analysis of the clot opens new perspectives for clot characterization. In perspective, correlations between 3D histopathology and microCT can provide quantification of clot composition in terms of CT density.

## Conclusion

Our study illustrates that microCT can be used for volumetric characterization of clots retrieved from acute ischemic stroke patients, for which both the intra-clot heterogeneity and inter-patient variability can be addressed.

MicroCT imaging emerges as a characterization technique which can bring new insights about the correlation between the human clots structure and clinical aspects in stroke.

## Data Availability Statement

The raw data supporting the conclusions of this article will be made available by the authors, without undue reservation.

## Ethics Statement

The study was approved, under the project ID 2018-00476, by the Cantonal Commission of Research Ethics (Commission cantonale d'éthique de la recherche, CCER) Geneva, who waived the requirement for written informed consent, in accordance with national legislation and institutional requirements.

## Author Contributions

All authors listed have made a substantial, direct, and intellectual contribution to the work and approved it for publication.

## Funding

The project has been funded by grants of the Swiss National Science Foundation (32003B_182382 and 320030_188942), by a grant of University Hospitals of Geneva, Radiology Department Startup fund, and a grant of the Science Foundation Ireland (13/RC/2073_2).

## Conflict of Interest

The authors declare that the research was conducted in the absence of any commercial or financial relationships that could be construed as a potential conflict of interest.

## Publisher's Note

All claims expressed in this article are solely those of the authors and do not necessarily represent those of their affiliated organizations, or those of the publisher, the editors and the reviewers. Any product that may be evaluated in this article, or claim that may be made by its manufacturer, is not guaranteed or endorsed by the publisher.

## Footnotes

^1^Available online at: http://www.slicer.org.
