# Supplementary material for: MicroCT Can Characterize Clots Retrieved With Mechanical Thrombectomy From Acute Ischemic Stroke Patients–A Preliminary Report
Source: Front Neurol. 2022 Mar 7;13:824091. doi: 10.3389/fneur.2022.824091 (PMC8934771; doi:10.3389/fneur.2022.824091)
Supplement: Supplementary file 1 [file Table_1.DOCX]

**Figure legends for Supplemental material:**

Figure S1 : Comparison microCT and histological staining: a – microCT slice of clot fixed in formalin; b – microCT slice of clot embedded in paraffin; c – histological staining of the clot with Martius Scarlet Blue (MSB); d – histological staining of the clot with hematoxylin and eosin (HE).

Figure S2 : Comparison microCT and histological staining: a – microCT slice of clot fixed in formalin; b – microCT slice of clot embedded in paraffin; c – histological staining of the clot with Martius Scarlet Blue (MSB); d – histological staining of the clot with hematoxylin and eosin (HE).

Figure S3 : MicroCT data can describe intra-clot heterogeneity. a) slice of a microCT scan performed for a formalin-fixed clot retrieved from a patient with acute ischemic stroke. b) segmentation performed for two density ranges (-817HU – to – 9 HU in green, 10 HU– to –178 HU in white) for the same clot scan, and c) to e) - maps computed based on textural features, using the BoneTexture module in Slicer (100 bins, neighborhood radius =4): c) GLCM energy map, d) GLCM entropy map, e) GLRM greyLevelNonUniformity map.

Figure S4 : Example of segmentation in a calcified white clot (which was not included in the pilot study).
