# Supplementary figures and images for: MicroCT Can Characterize Clots Retrieved With Mechanical Thrombectomy From Acute Ischemic Stroke Patients–A Preliminary Report
Source: Front Neurol. 2022 Mar 7;13:824091. doi: 10.3389/fneur.2022.824091 (PMC8934771; doi:10.3389/fneur.2022.824091)

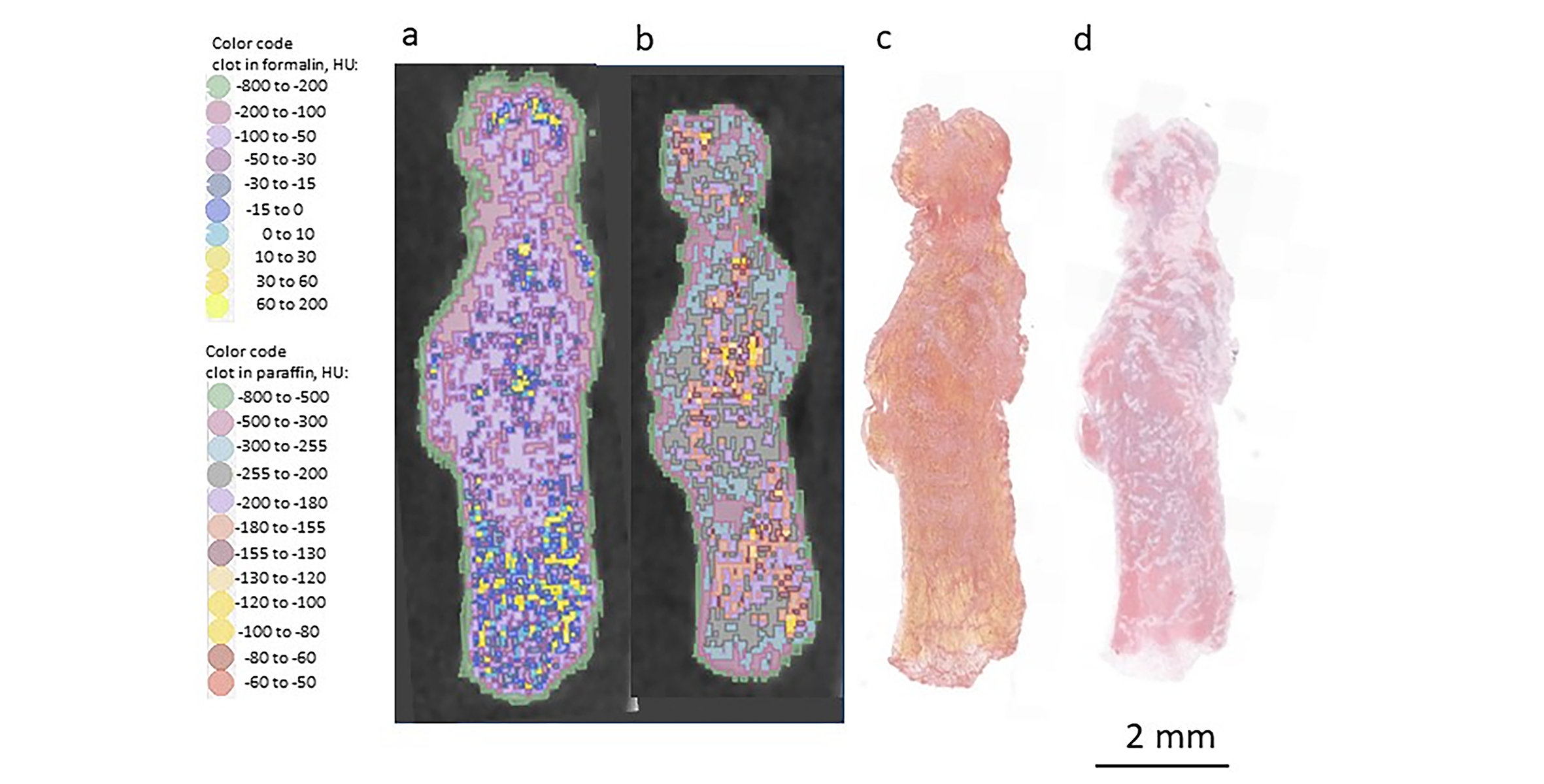

Supplement: Supplementary file 2 [file Image_1.TIF]

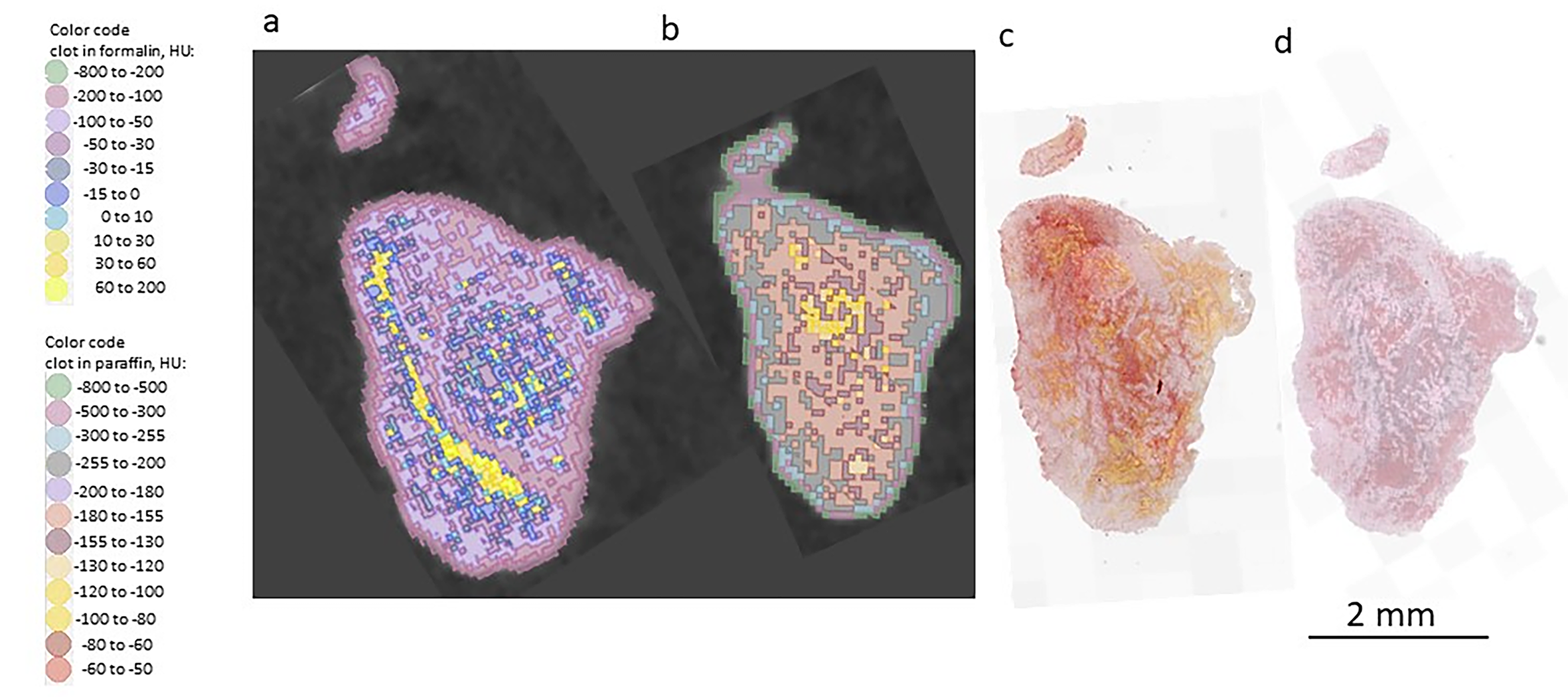

Supplement: Supplementary file 3 [file Image_2.TIF]

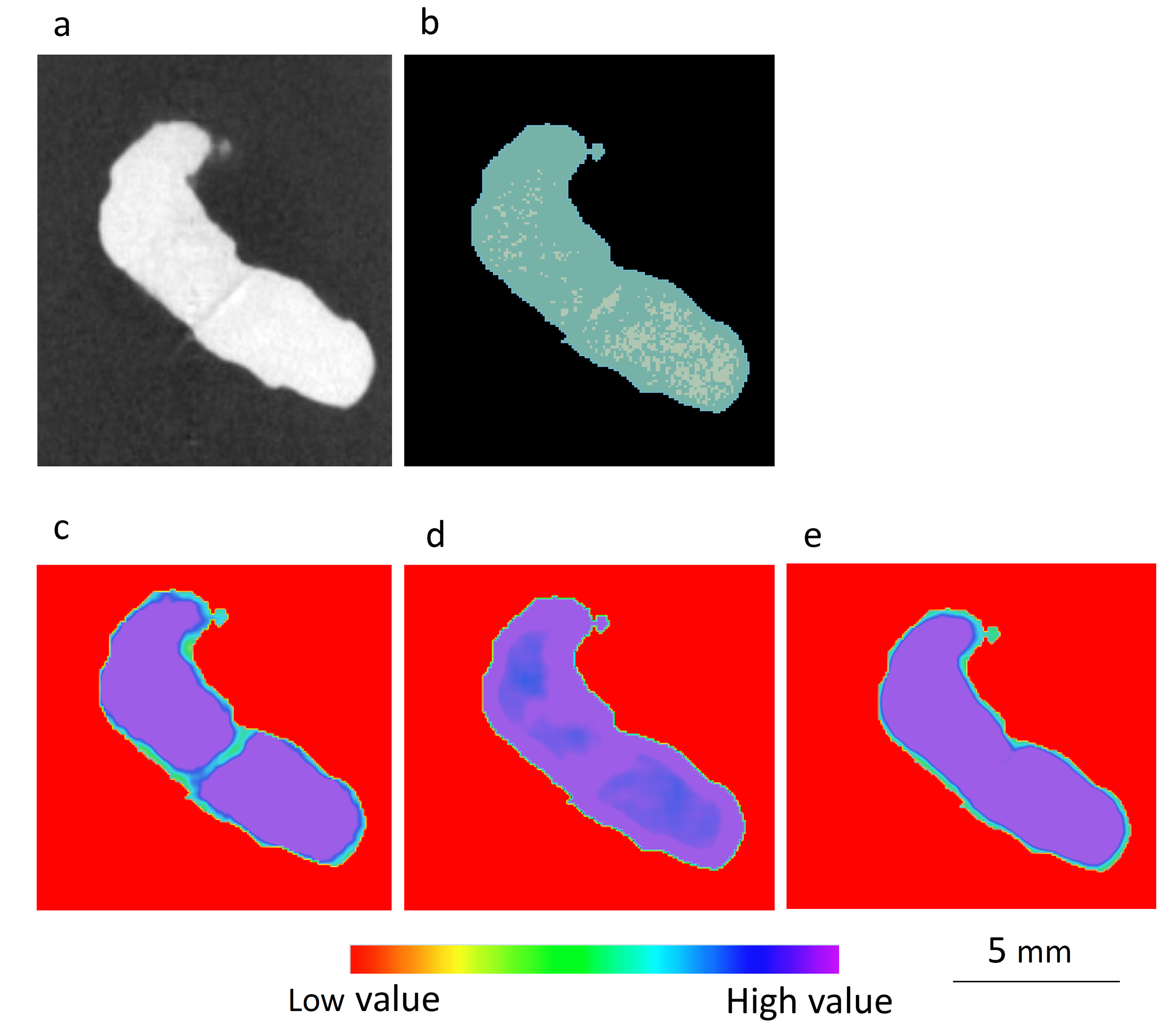

Supplement: Supplementary file 4 [file Image_3.TIF]

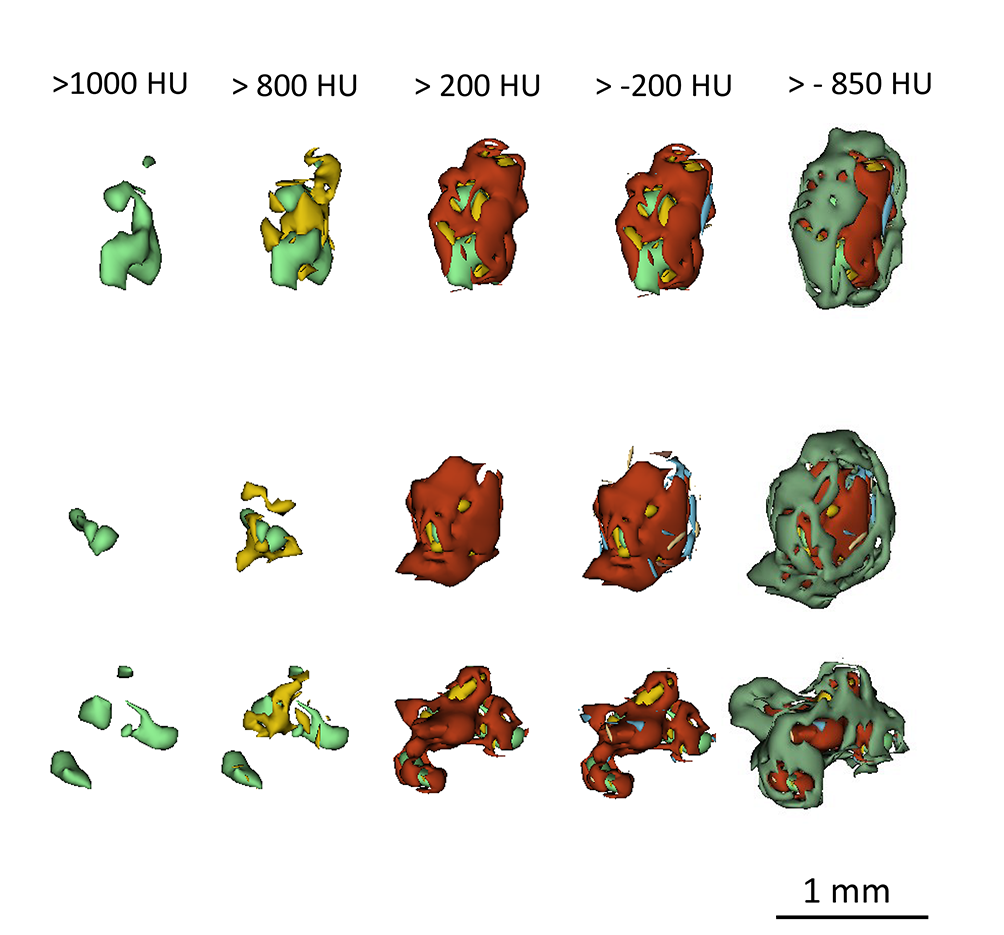

Supplement: Supplementary file 5 [file Image_4.TIF]
